# Polyubiquitin Is Required for Growth, Development and Pathogenicity in the Rice Blast Fungus *Magnaporthe oryzae*

**DOI:** 10.1371/journal.pone.0042868

**Published:** 2012-08-10

**Authors:** Yeonyee Oh, William L. Franck, Sang-Oh Han, Angela Shows, Emine Gokce, David C. Muddiman, Ralph A. Dean

**Affiliations:** 1 Center for Integrated Fungal Research, Department of Plant Pathology, North Carolina State University, Raleigh, North Carolina, United States of America; 2 Department of Medicine, Duke University, Durham, North Carolina, United States of America; 3 W. M. Keck FT-ICR Mass Spectrometry Laboratory, Department of Chemistry, North Carolina State University, Raleigh, North Carolina, United States of America; University of Minnesota, United States of America

## Abstract

Protein ubiquitination, which is highly selective, regulates many important biological processes including cellular differentiation and pathogenesis in eukaryotic cells. Here, we integrated pharmacological, molecular and proteomic approaches to explore the role of ubiquitination in *Magnaporthe oryzae*, the leading fungal disease of rice world-wide. Inhibition of ubiquitin-mediated proteolysis using the 26S proteasome inhibitor, Bortezomib, significantly attenuated conidia germination, appressorium formation and pathogenicity in *M. oryzae*. Gene expression analysis revealed that many genes associated with protein ubiquitination were developmentally regulated during conidia germination. Only a few, including a polyubiquitin encoding gene, MGG_01282, were more abundantly expressed during appressorium formation and under nitrogen starvation. Targeted gene deletion of MGG_01282, in addition to a significant reduction in protein ubiquitination as determined by immuno blot assays, resulted in pleiotropic effects on *M. oryzae* including reduced growth and sporulation, abnormal conidia morphology, reduced germination and appressorium formation, and the inability to cause disease. Mutants were also defective in sexual development and were female sterile. Using mass spectrometry, we identified 63 candidate polyubiquitinated proteins under nitrogen starvation, which included overrepresentation of proteins involved in translation, transport and protein modification. Our study suggests that ubiquitination of target proteins plays an important role in nutrient assimilation, development and pathogenicity of *M. oryzae*.

## Introduction

Ubiquitin mediated protein degradation is a highly conserved process and plays important roles in a variety of cellular processes, including transcriptional regulation, signal transduction, cell cycling, cellular differentiation and pathogenesis [1]. Ubiquitin, a highly conserved 76-amino acid protein, is activated by the ubiquitin activating enzyme E1 using ATP, which is then transferred to a ubiquitin conjugating enzyme E2. The E2 enzyme and the protein substrate bind specifically to a particular ubiquitin-protein ligase E3 resulting in the carboxy-terminal glycine of ubiquitin becoming covalently attached to a lysine residue of the protein substrate through an iso-peptide bond. The specificity of targeted proteins is largely controlled by E3 ligases. Successive conjugation of ubiquitin generates a polyubiquitin chain that is recognized by regulatory particles in the proteasome for degradation or as a trigger for various signaling pathways [1].

Ubiquitin is one of the most abundant cellular proteins, representing 1–5% of total cellular protein [2], [3]. Ubiquitin levels are maintained by recycling of ubiquitin from ubiquitin substrate conjugates by deubiquitinating enzymes (DUBs) and de novo ubiquitin synthesis through transcriptional regulation. In yeast, ubiquitin is produced by cleavage from precursor proteins, where ubiquitin is fused to unrelated peptide sequences (*UBI1, UBI2 and UBI3* ) or to itself (*UBI4*) [4], [5]. *UBI4* encodes a polyubiquitin protein that contains five consecutive ubiquitin repeats and is highly induced under stress conditions [4], [5]. Ubiquitination appears to play an important role in host-pathogen interactions. In a number of plant pathogenic fungi, polyubiquitin transcript levels significantly increased during *in planta* colonization or under environmental stress [6]–[8]. In the human pathogen *Candida albicans*, inactivation of polyubiquitin gene *UBI4* affected fungal growth, stress resistance and virulence [9]. However, direct evidence for the role of ubiquitination in plant pathogenic fungi is lacking.

Rice blast is the most important disease of rice worldwide, and is caused by the filamentous ascomycete fungus, *Magnaporthe oryzae* [10]. Common to many other phytopathogenic fungi, *M. oryzae* elaborates a specialized infection cell, the appressorium to infect its host [11]. Perception of environmental cues [12], starvation responses [13], cell signaling pathways [12], [14], [15], turgor pressure generation [16], recycling cellular contents (autophagy) [17] and cell cycle checkpoints [18] are known to orchestrate the development of this specialized cell. Through global gene expression and functional analyses, we previously identified a connection between protein turnover and the infection process. Genes including *SPM1*, a vacuolar protease and *MGD1*, a putative NAD(+) dependent glutamate dehydrogenase (required for recycling carbon and nitrogen from amino acids back into metabolism) were up regulated during appressorium formation. Deletion of either gene resulted in defective appressoria and greatly reduced ability to cause disease [19]. We also observed the transcripts of polyubiquitin gene (MGG_01282) to be significantly enriched during appressorium development as well as under nitrogen starvation conditions [13], [19]. In this study, we conducted a comprehensive investigation of the role of protein ubiquitination in *M. oryzae* using pharmacological, molecular and proteomic approaches. Our findings suggest that ubiquitin-mediated proteolysis, which is known to be highly selective, plays a key role in nutrient assimilation, fungal development and pathogenicity of *M. oryzae*.

## Materials and Methods

### Effect of a Proteasome Inhibitor, Bortezomib, on M. oryzae

Wild type *M. oryzae* strain 70-15 was used for all experiments unless otherwise indicated. Conidia were collected from solidified V8 medium after 8 days in water and adjusted to 5×10^4^ conidia/ml. Bortezomib (LC laboratories) stock was prepared in DMSO (4 ug/ul) and added to the conidial suspension to a bring the final concentration up to 50 uM. 50 ul of conidial suspension was placed on the appressorium inducing hydrophobic surface of Gelbond film (Lonza). After 24 and 48 hr incubation, conidia germination and appressorium formation were assessed. For infection assays, 10 ul of conidial suspension in Bortezomib was spotted onto detached 6 day old barley (ROBUST) leaves, placed in a humid plastic container and disease progress monitored for 5 days. Experiments were repeated three times with 3 replicates.

**Figure 1:**
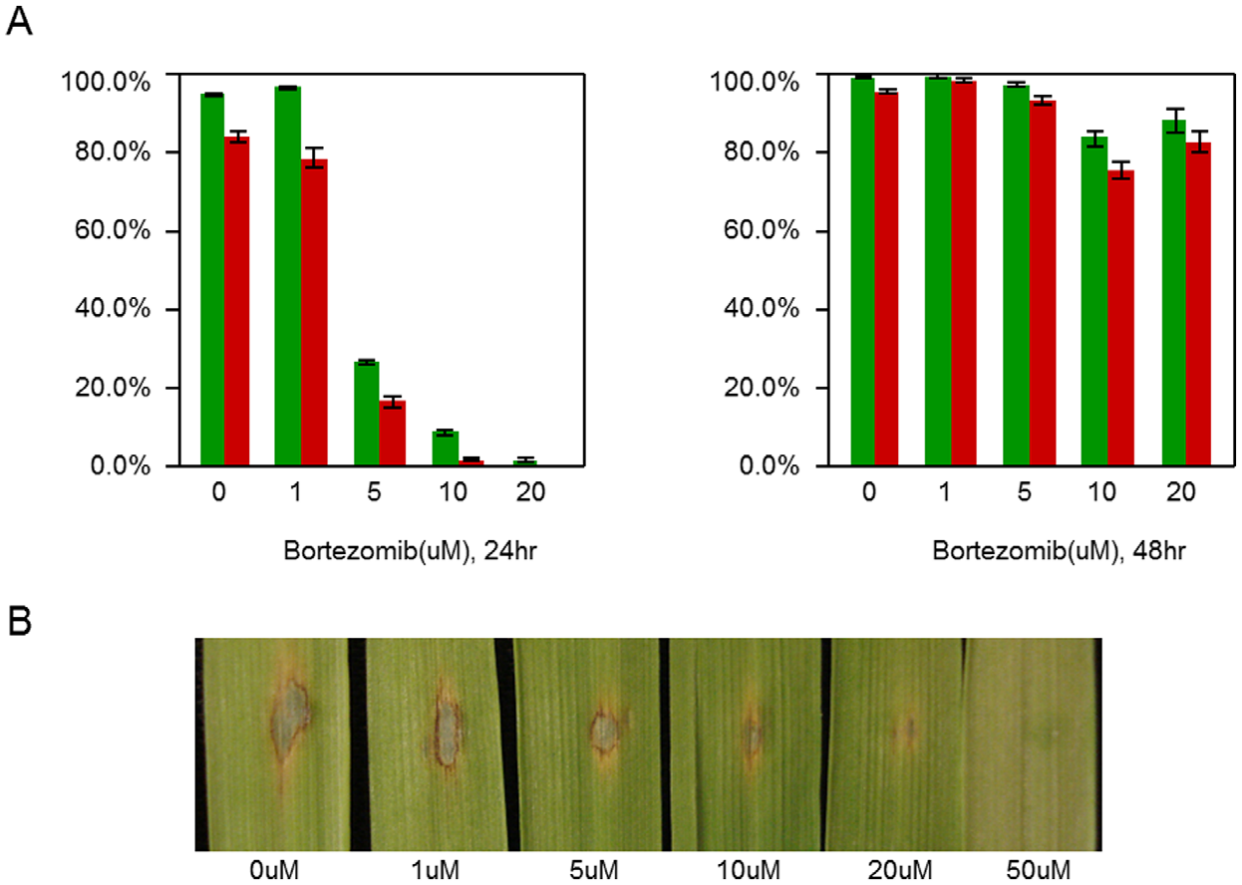
Bortezomib blocks conidia germination, appressorium formation and pathogenicity. A. Conidia germination and appressorium formation (green and red bar respectively) were measured after 24 and 48 hr incubation on a hydrophobic surface. B. Pathogenicity assays were performed on 6 day old barley leaves. Disease progress was assessed after 5 day incubation.

### Prediction of Ubiquitin Associated Proteins in M. oryzae and their Transcription Analysis

Ubiquitin associated (UA) InterPro domains were selected based on other studies [20]. *M. oryzae* proteins with UA Interpro domains were extracted from European Bioinformatics Institute (EBI) Interpro databases (http://www.ebi.ac.uk/interpro/) using the Biomart filtering tool and were categorized according to function in the ubiquitination pathway. Gene expression for UA proteins during fungal development and nitrogen starvation were extracted from the previously reported genome-wide *M. oryzae* microarray data (NCBI GenBank: GSE1945, GSE10173 and GSE 2716).

**Table 1:** Ubiquitin pathway associated proteins in *M. oryzae*.

Class		InterPro domain		# of proteins
**UA**		IPR000626	Ubiquitin	12
		IPR004854	Ubiquitin fusion degradation protein UFD1	2
		IPR001012	UBX	6
		IPR003892	Ubiquitin system component Cue	6
		IPR000449	Ubiquitin-associated/translation elongation factor EF1B, N-terminal	7
		IPR003903	Ubiquitin interacting motif	13
	UA total			44
**E1**		IPR000011	Ubiquitin-activating enzyme, E1-like	2
		IPR000127	Ubiquitin-activating enzyme repeat	3
		IPR000594	UBA/THIF-type NAD/FAD binding fold	8
	E1 total			8
**E2**		IPR000608	Ubiquitin-conjugating enzyme, E2	21
	E2 total			21
**E3**		IPR001232	SKP1 component	1
		IPR003126	Zinc finger, N-recognin	2
		IPR003613	U box domain	3
		IPR001373	Cullin, N-terminal	4
		IPR000569	HECT	6
		IPR001810	F-box domain, cyclin-like	26
		IPR001841	Zinc finger, RING-type	53
	E3 total			94
**DUB**		IPR001607	Zinc finger, UBP-type	3
		IPR001578	Peptidase C12, ubiquitin carboxyl-terminal hydrolase 1	4
		IPR001394	Peptidase C19, ubiquitin carboxyl-terminal hydrolase 2	15
	DUB total			20
Total				183

### Targeted Gene Replacement and Complementation of MGG_01282

Gene replacement cassettes were constructed using adaptamer mediated PCR as previously described [21]. Briefly about 1 kb of upstream and downstream sequence of MGG_01282 gene was amplified with primers that contained adaptamer sequences (see Table S1). A 1.5 kb fragment containing the hygromycin B phosphotransferase gene (HPH) driven by the *trp*C promoter from *Aspergillus nidulans* was amplified from plasmid PCB1003 using the adaptamer sequence attached to the forward HPHF and reverse HPHR primer set. Using nested primers (see Table S1) from inside of the 5′ upstream fragment and from inside of the 3′ end of the downstream fragment of the target gene, the individual fragments and hygromycin resistance gene fragment were combined and amplified together to construct a hygromycin cassette for gene replacement approximately ∼3.1 kb in length. The hygromycin cassette was transformed into 70-15 protoplasts as previously described [22]. Gene replacement mutants were identified by PCR screening and further confirmed by Southern blot analysis using ECL system of oligolabeling and detection (Amersham Co.) (Figure S1). For complementation, a 2.6 kb DNA fragment corresponding to the MGG_01282 gene and its promoter region was PCR amplified (see Table S1) from 70-15 wild type genomic DNA, and using gateway cloning system was cloned into a modified pDONR221 plasmid in which the Bialophos resistance gene had been inserted. The complementation construct was introduced into mutant protoplasts using standard protocols and transformants were screened on Bialophos at 200 µg/ml. The complemented strains were identified by PCR amplification of the insert and further confirmed by the recovery of wild type phenotypes.

**Figure 2:**
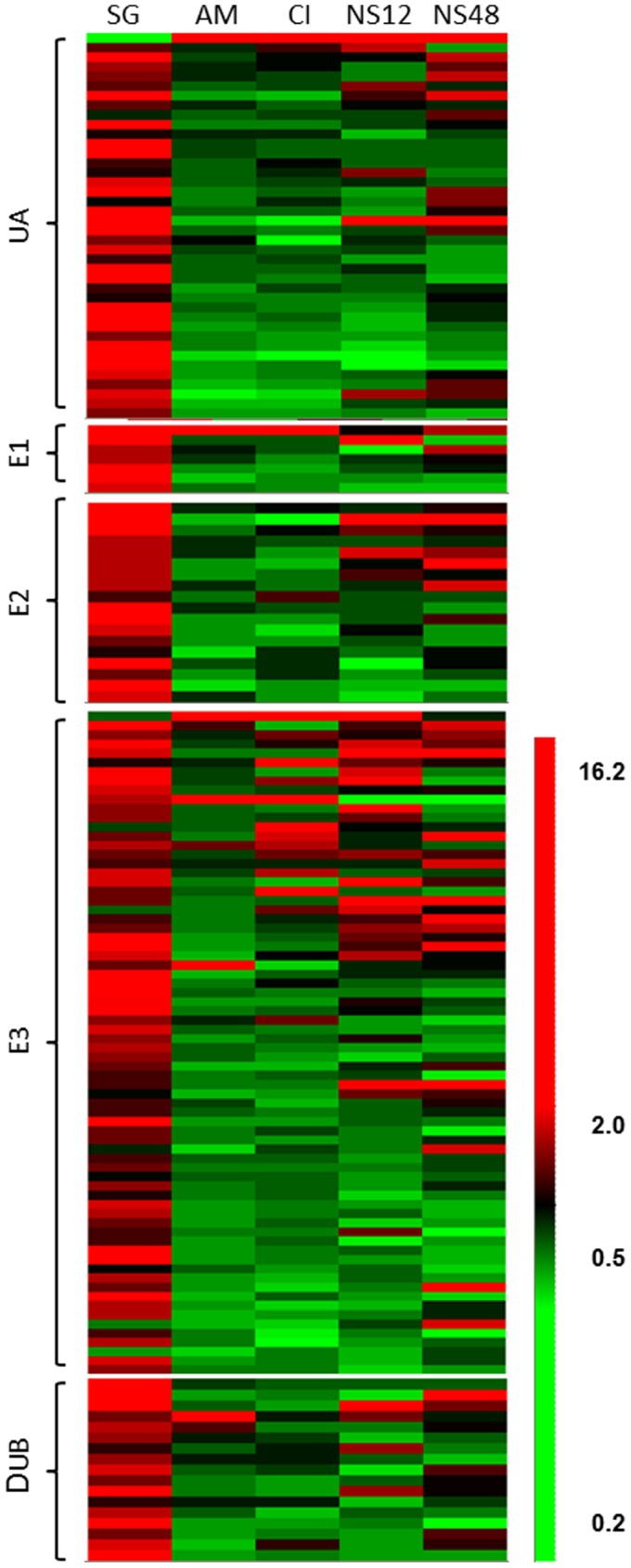
Ubiquitin associated genes are differentially expressed during conidia germination, appressorium formation and under nitrogen starvation. Each column represents hierarchical clustering profile for spore germination (SG), appressorium maturation (AM), cAMP induced appressoria formation (CI) and nitrogen starvation for 12 (N12) and 48 (NS48) hr. Genes of same expected function were grouped together as ubiquitin associated (UA), ubiquitin-activating enzymes (E1), ubiquitin-conjugating enzymes (E2), ubiquitin protein ligases (E3) and de-ubiquitinating enzymes (DUB).

### Mutant Phenotype Assays

A series of phenotype analyses were conducted on several knockout mutants (≥3) and ectopic (≥2) transformants. Germination and appressorium assays were conducted using conidia collected from 8 day old V8 agar plates and adjusted to 5×10^4^ conidia/ml. Conidia suspension was spotted on the hydrophobic and hydrophilic surface of GelBond film and rate of germination and appressorium formation was measured after 24 hr incubation at 25°C in the dark. To test for pathogenicity, barley and rice seedlings were spray inoculated with *M. oryzae* conidia suspension (5×10^4^ conidia/ml, Tween 20 0.025%) and incubated in dark humid conditions at 25°C. The number and size of lesions were evaluated 5 days post-inoculation. Lesions on three leaves were counted for each strain, and this experiment was repeated three times. Wound assays were performed by a pinprick with a sterile needle on detached barley leaves. 5 millimeter square agar blocks from 8 day old culture on V8 of each strain were directly placed onto the wound site, and incubated in humid chambers as described above. Measurements were taken from nine wound sites (three per leaf) per strain, and this experiment was repeated twice. Disease progress and symptom development of MGG_01282 mutant was compared to wild type, ectopic, complemented strain or control treatment. Growth rate assays were conducted by placing 10 ul conidia suspension (5×10^4^ conidia/ml) on agar plates with complete media, minimal media and minimal media without nitrogen source. Colony morphology and diameters were recorded periodically for 15 days. The total number of conidia on minimal media plates was counted after 15 days incubation. Conidia size and morphology were also assessed using a minimum of 100 conidia per replicate. All experiments were conducted in triplicate and performed at least 3 times.

**Figure 3:**
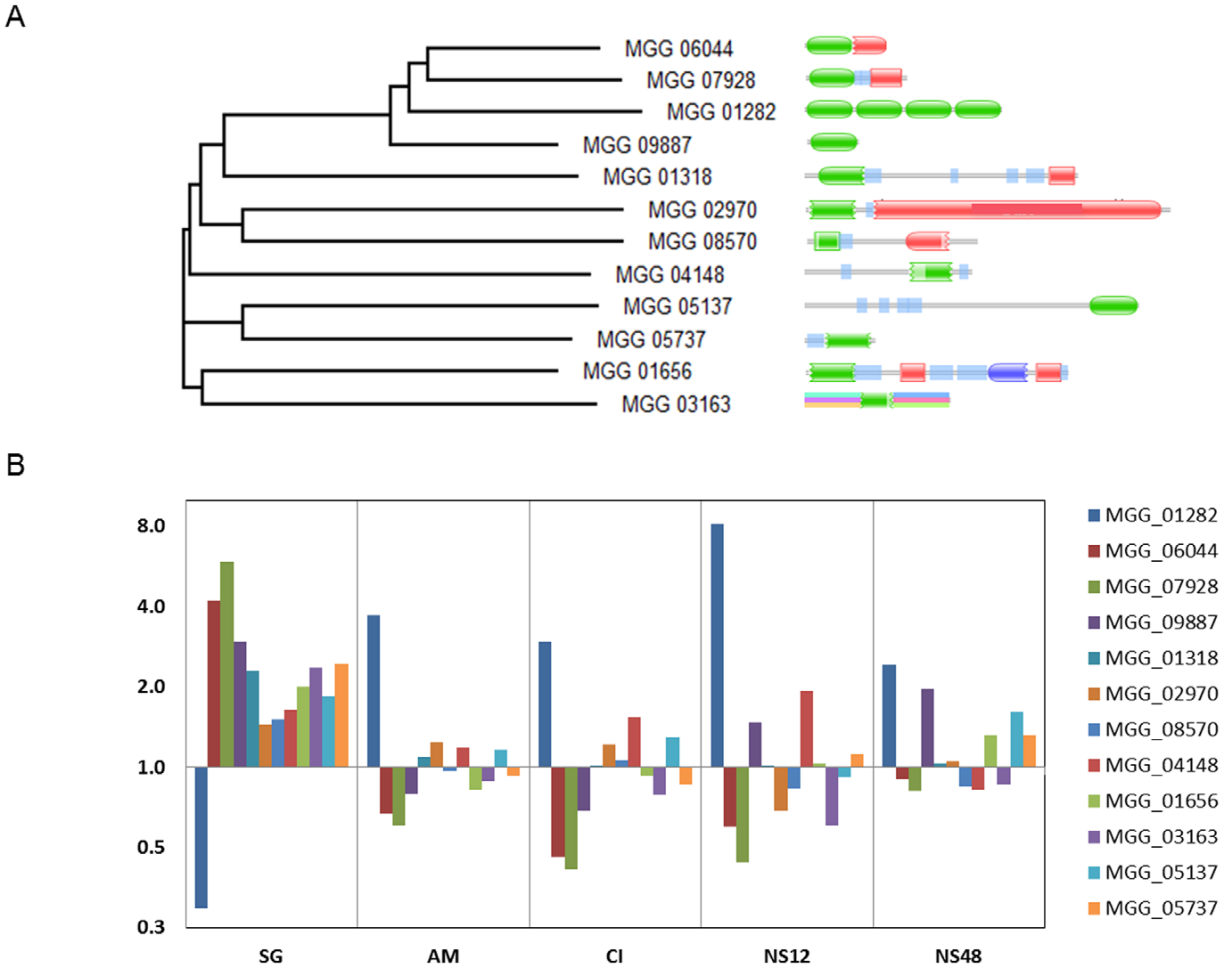
Clustering and gene expression of 12 ubiquitin related proteins in *M. oryzae*. A. Proteins were grouped using ClustalW and functional domains indicated. Green oval structure indicates a ubiquitin moiety and others in green indicate partial or ubiquitin like domains. B. Gene expression during spore germination (SG), appressorium maturation (AM), cAMP induced appressoria formation (CI ) and nitrogen starvation for 12 (NS12) and 48 (NS48) hr.

### Sexual Reproduction

*M*. *oryzae* 70–15 strains were crossed with *M. oryzae* strain 4091-5-8, a weeping lovegrass (*Eragrostis curvula*) pathogen [23] on oatmeal media. Plates were incubated at 25°C for 7 days and then incubated at 20°C for 21 days under constant light. Plates were examined for fruiting body formation. Perithecia were excised and crushed to identify ascospores under a brightfield microscope.

**Figure 4:**
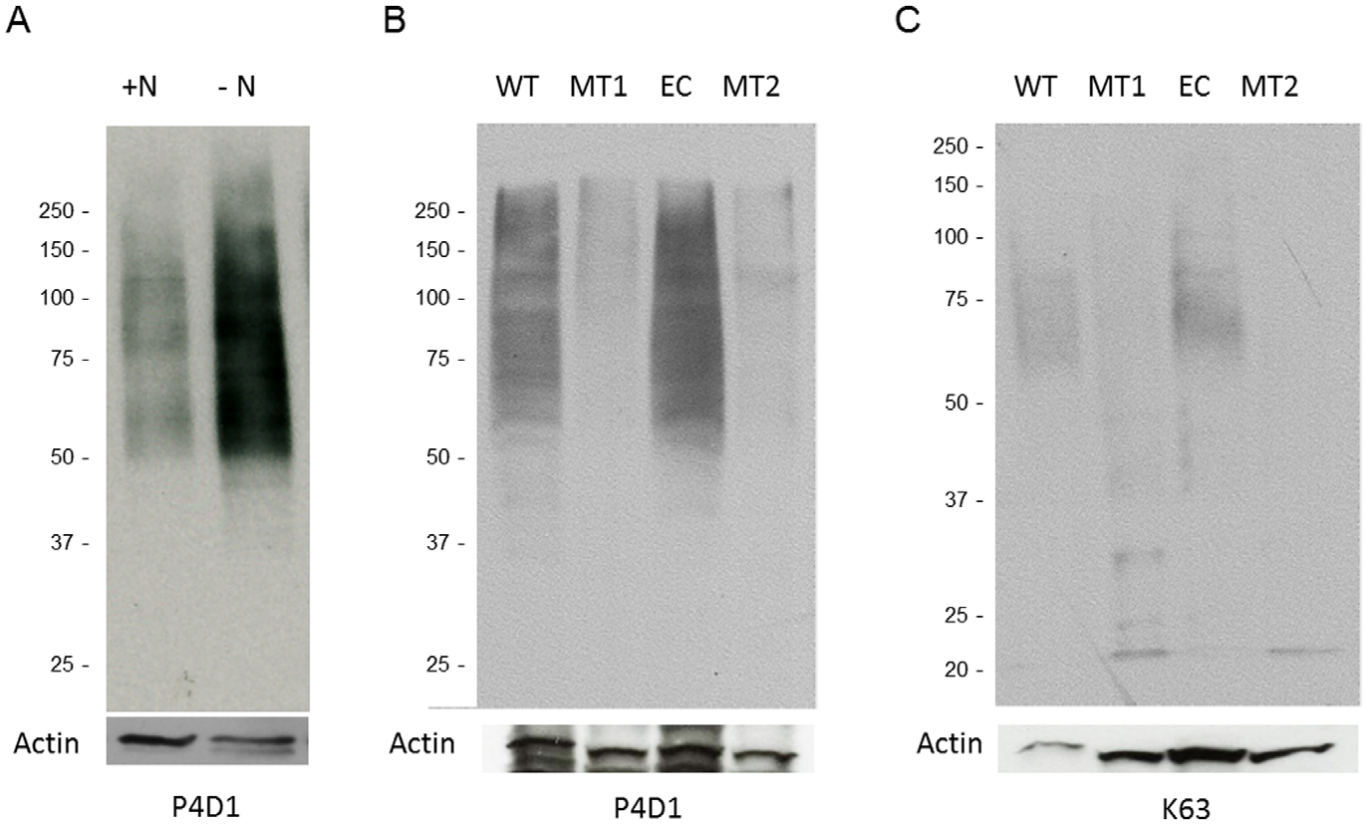
Protein ubiquitination is induced by nitrogen starvation and requires the polyubiquitin gene, MGG_01282. A. 70-15 *M. oryzae* was grown in liquid minimal medium with (+N) and without (−N) nitrogen sources. Protein extracts from each sample were probed with an antibody recognizing both ubiquitin and polyubiquitin (P4D1). An anti-actin antibody was used to compare the relative amount of total proteins in each lane. B and C. Wild type (WT), MGG_1282 deletion mutants (MT1, MT2) and ectopic (EC) strains were incubated in minimal medium. Protein extracts from each sample were probed with antibodies recognizing ubiquitin and polyubiquitin (P4D1) or specifically polyubiquitin (K63), respectively. Panel B were exposed to X ray film for a longer compared to panel A.

### Western Blotting

Mycelia samples from wild type, mgg_01282 mutant and ectopic strains were collected from 3 day old liquid minimal media cultures, filtered through miracloth and washed with water. Excess liquid was squeezed out and 500 mg of semi-dried mycelia was ground using liquid nitrogen and resuspended with 2 ml lysis buffer containing 50 mM HEPES (pH 7.5), 0.5% Nonidet P-40, 250 mM NaCl, 10% (v/v) glycerol, 2 mM EDTA (pH 8.0), and a complete protease inhibitor cocktail (Roche). Protein quantification of each soluble lysate was performed by Bradford assay. 85 ug of each protein sample was separated on a 4–20% gradient gel (Invitrogen) and transferred to a nitrocellulose membrane. Blots were blocked in 5% bovine serum albumin (BSA) and antibody incubations were carried out in 5% skim milk followed by washes. Signals were detected by SuperSignal West Pico Chemiluminescent Substrate (Pierce), following the manufacturer’s instructions. The following antibodies were used. P4D1 (1∶1000, Cell Signaling Technology), K63 (1∶1000, Cell Signaling Technology) and Anti-mouse IgG (1∶3000, Cell Signaling Technology).

**Figure 5:**
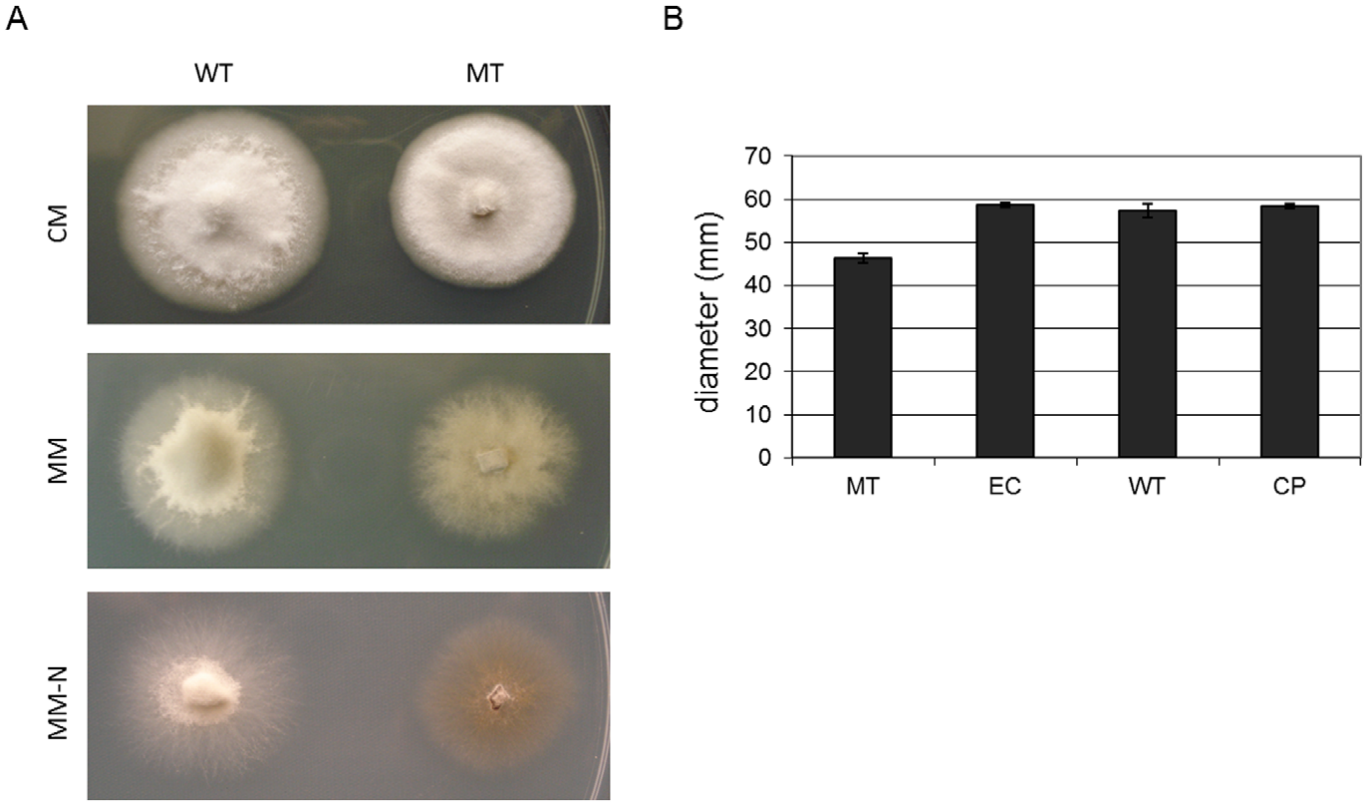
MGG_01282 is required for normal vegetative growth. A. 70-15 wild type (WT) and mutant strain (MT) were incubated for 7 days on complete medium (CM), minimal medium (MM) and minimal medium without nitrogen source (MM-N). B. Radial mycelial growths were measured in mutant stain MT1 and MT2 and were compared to those of ectopic (EC), WT and MGG_012982 complemented strain (CP).

### Ubiquitinated Protein Profiling by NanoLC-MS/MS

Wild type *M. oryzae* 70-15 was incubated in liquid complete media for 3 days. Mycelia were collected, washed thoroughly with distilled water and then inoculated into liquid minimal media without nitrogen source. After 12 hr incubation, 3 replicates were pooled, mycelia tissues were collected and proteins extracted. The putative polyubiquitinated protein sample was enriched using Agarose-Tube2 (LIfeSensors) following manufacturer’s protocol. To prepare the sample for NanoLC-MS/MS, dithiothreitol (Biorad, Hercules) was added to the protein sample to a final of 5 mM and was incubated for 30 min at 56°C to reduce the protein disulfide bonds. The samples were then mixed with 200 µl of 8 M Urea (Sigma Aldrich), loaded onto Vivacon 500 µL ultrafiltration spin columns with 30 kDa MW cutoff (Sartorius Stedim Biotech) and centrifuged at 14,000×g for 15 min. The columns were washed one more time before Iodoacetamide (Sigma Aldrich) was added to a final of 20 mM. The samples were incubated for 30 min in the dark at room temperature for alkylation of the free thiols. The filter units were then centrifuged at 14,000×g for 10 min. Three 100 µl 8 M Urea washes and 3 0.05 M ammonium bicarbonate (Sigma Aldrich) washes with 15 min subsequent centrifugations were performed. The collection vials were changed and 0.4 µg/µl trypsin (Sigma Aldrich) was added at a 1∶100 enzyme: protein ratio. Digestion was performed over night at 37°C and peptides eluted by adding 40 µl ammonium bicarbonate and centrifuging the filter units at 14,000×g for 10 min. Each sample was injected 3 times to a LTQ FT Ultra Hybrid Mass Spectrometer and the data analyzed as previously described [24].

**Figure 6:**
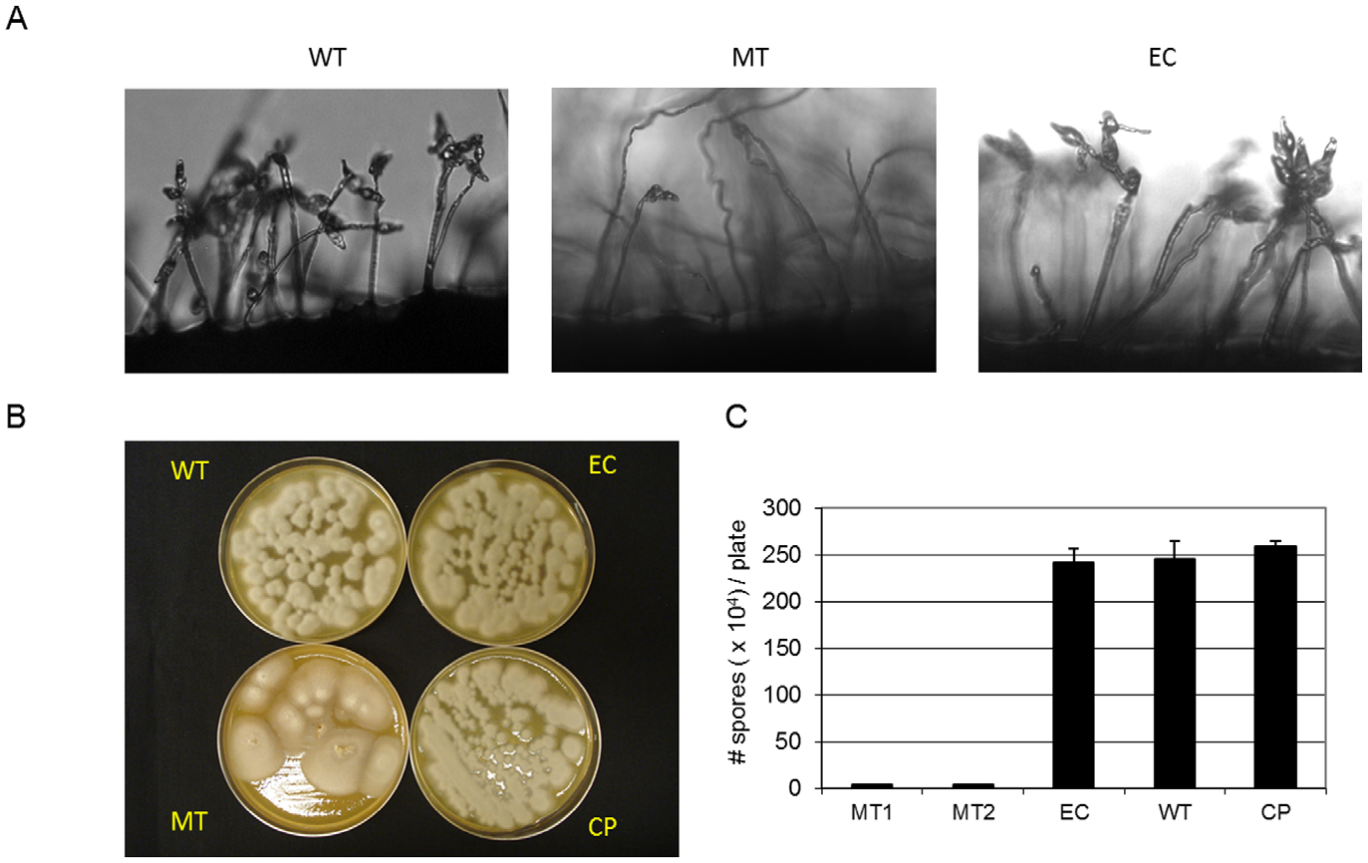
MGG_01282 is required for normal conidia production. 70–15 wild type (WT), MGG_01282 deletion mutants (MT1, MT2), ectopic (EC) and MGG_01282 complemented (CP) strains were incubated on V8 medium (A, B) and the average number of conidia produced 10 days after inoculation (C).

**Figure 7:**
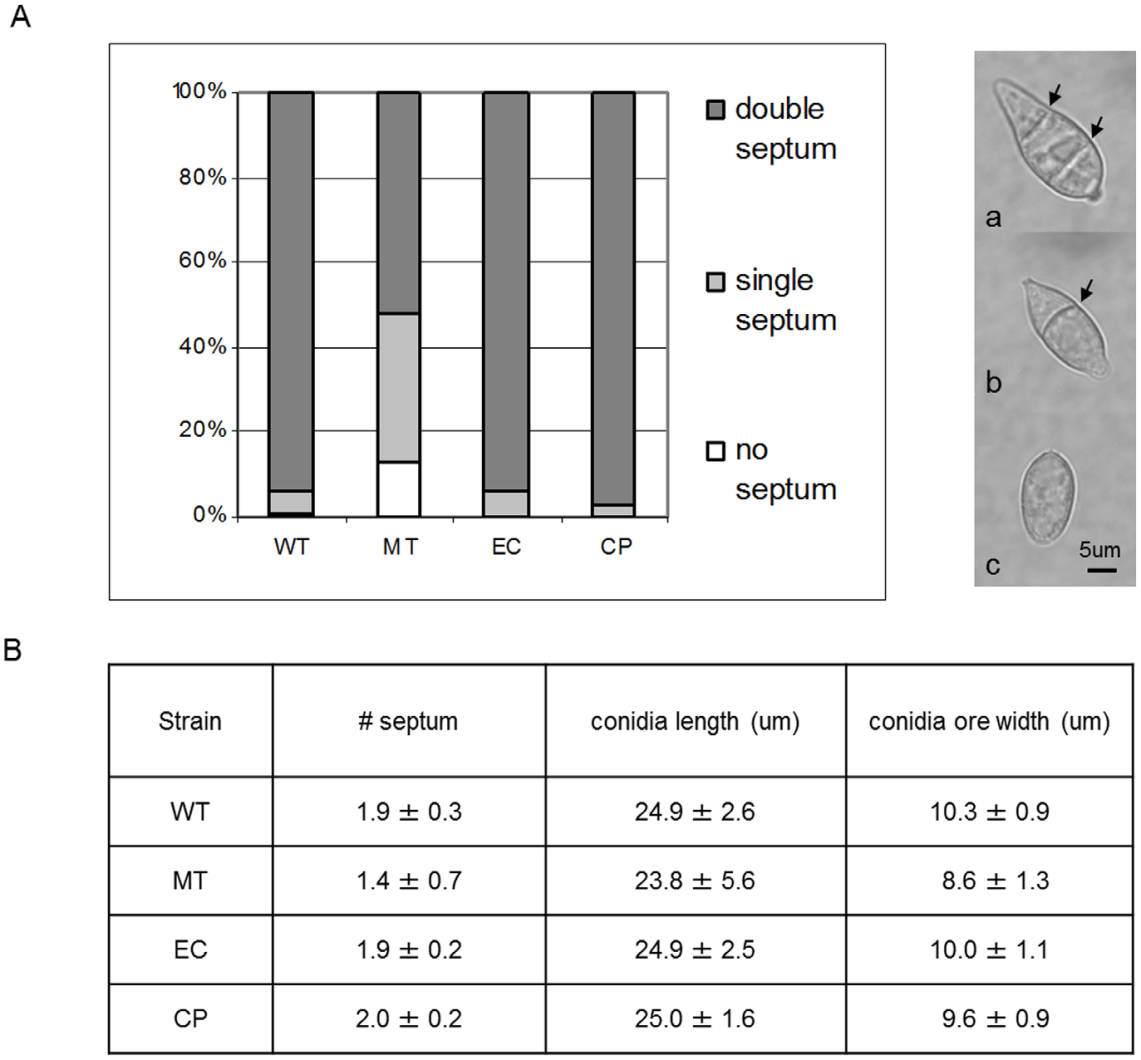
MGG_01282 deletion mutants produced abnormal conidia. A. For each strain, conidia collected from V8 medium were grouped according to the number of septa, double (a); single (b) or no septa (arrow marked). The percentage of each group per strain is presented. B. Average number of septa, conidia length and width is presented.

## Results

### Inhibition of Proteasome Mediated Protein Degradation Blocks Conidial Germination, Appressorium Induction and Pathogenicity in M. oryzae

To investigate the role of ubiquitin mediated proteasomal protein degradation during the infection-related fungal development, we treated conidia with the proteasome inhibitor, Bortezomib. On an appressorium inductive hydrophobic surface, both conidial germination and appressorium formation were significantly delayed in a dose-dependent manner with 20 uM Bortezomib being completely inhibitory after 24 hr incubation. However, at 48 hr, most conidia germinated and successfully formed melanized appressoria (Figure 1A, 1B). Bortezomib also inhibited pathogenicity. Compared to typical spreading necrotic lesions on control infected barley leaves, the addition of 1 uM Bortezomib resulted in smaller lesions. Symptom development was completely blocked when the conidia solution contained 50 uM Bortezomib (Figure 1C). 50 uM Bortezomib solution had no observable effect on barley leaves. These data are consistent with proteasome mediated protein turnover being required for infection related development and pathogenicity in *M. oryzae*.

**Figure 8:**
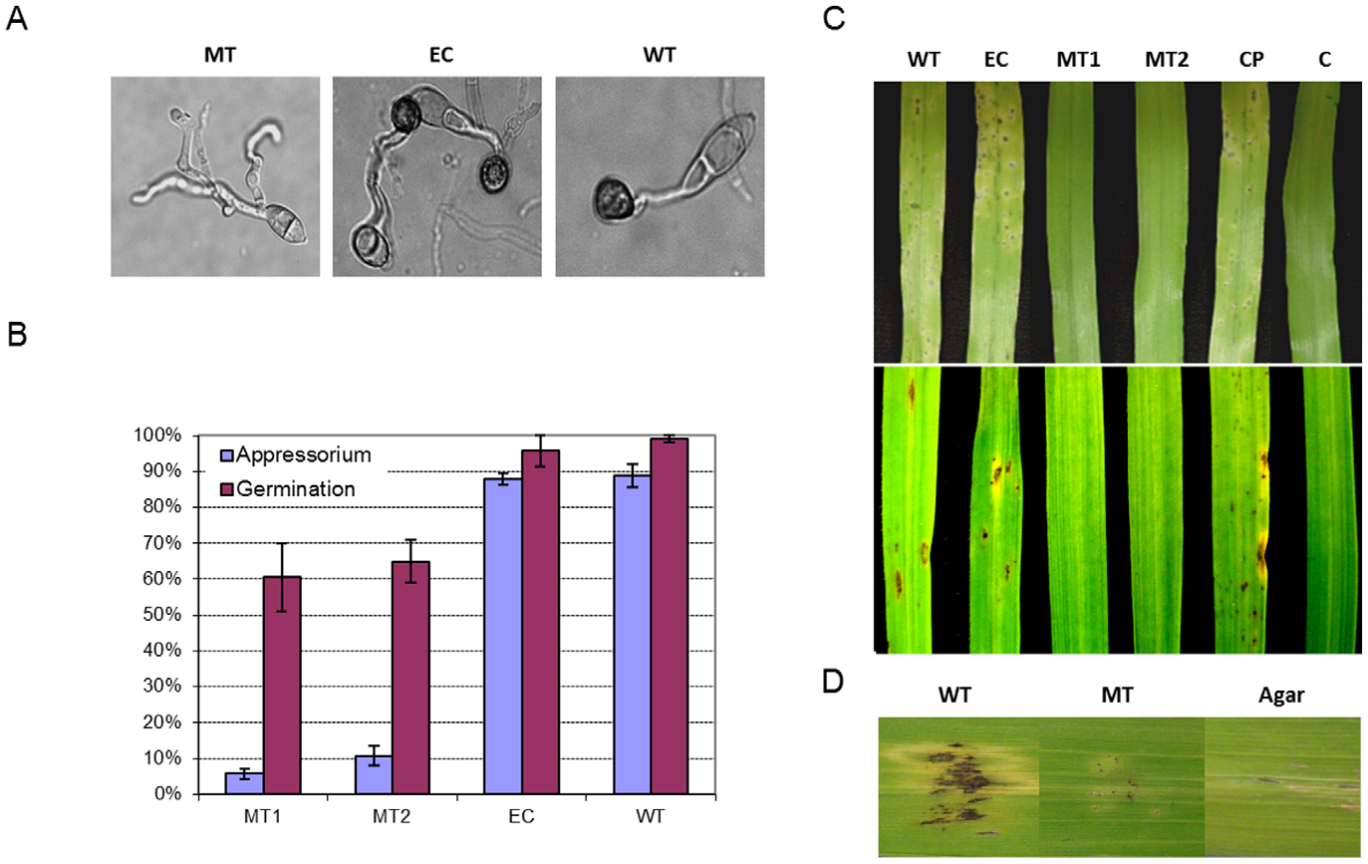
MGG_01282 is required for appressorium formation and pathogenicity in rice and barley. A. Germination and appressorium formation of conidia from 70-15 wild type (WT), MGG_01282 deletion mutants (MT1, MT2) and ectopic (EC) strains on a hydrophobic surface after 24 hr incubation. B. Percentage of conidia germination and appressorium formation from at least 100 conidia per replicate with 3 replicates per strain. C. Disease development of each strain including a complemented (CP) strain inoculated onto barley (upper panel) and rice (lower panel) seedlings. Disease progress was evaluated compared to water treated control (C) 5 days after inoculation. D. Wound assay on detached barley leaves. Barley leaves were wounded by making a tiny pinprick with a sterile needle. Leaves were inoculated with 8 day old V8 agar block of each strain and incubated in a humid chamber. Disease progress was evaluated compared to control agar and photographed 5 days post-inoculation.

### Components of Ubiquitin Mediated Protein Modification are Highly Conserved and Regulated in Response to Developmental and Nutritional Stimuli in M. oryzae

To assess the degree of conservation of proteins associated with ubiquitin mediated protein modification, we conducted an InterPro domain search. One hundred and eighty three proteins putatively involved in ubiquitination pathway in *M. oryzae* were identified (Table 1, Table S2). Forty four proteins directly related to ubiquitin (UA) were found including 12 containing ubiquitin and 13 with an ubiquitin interacting motif. Eight proteins were ubiquitin-activating enzymes, E1, and 21 proteins were ubiquitin-conjugating enzymes, E2. The most diverse and largest group was ubiquitin ligase, E3, which had 94 members and included 53 zinc finger, ring-type (IPR001841) domain containing proteins. Components of multi-protein E3 ubiquitin ligase Skp, Cullin and F-box containing complex (SCF complex) were found and included one Skp, four Cullins and 26 F-box containing proteins. A total of 20 proteins were identified as proteins for de-ubiquitination.

**Figure 9:**
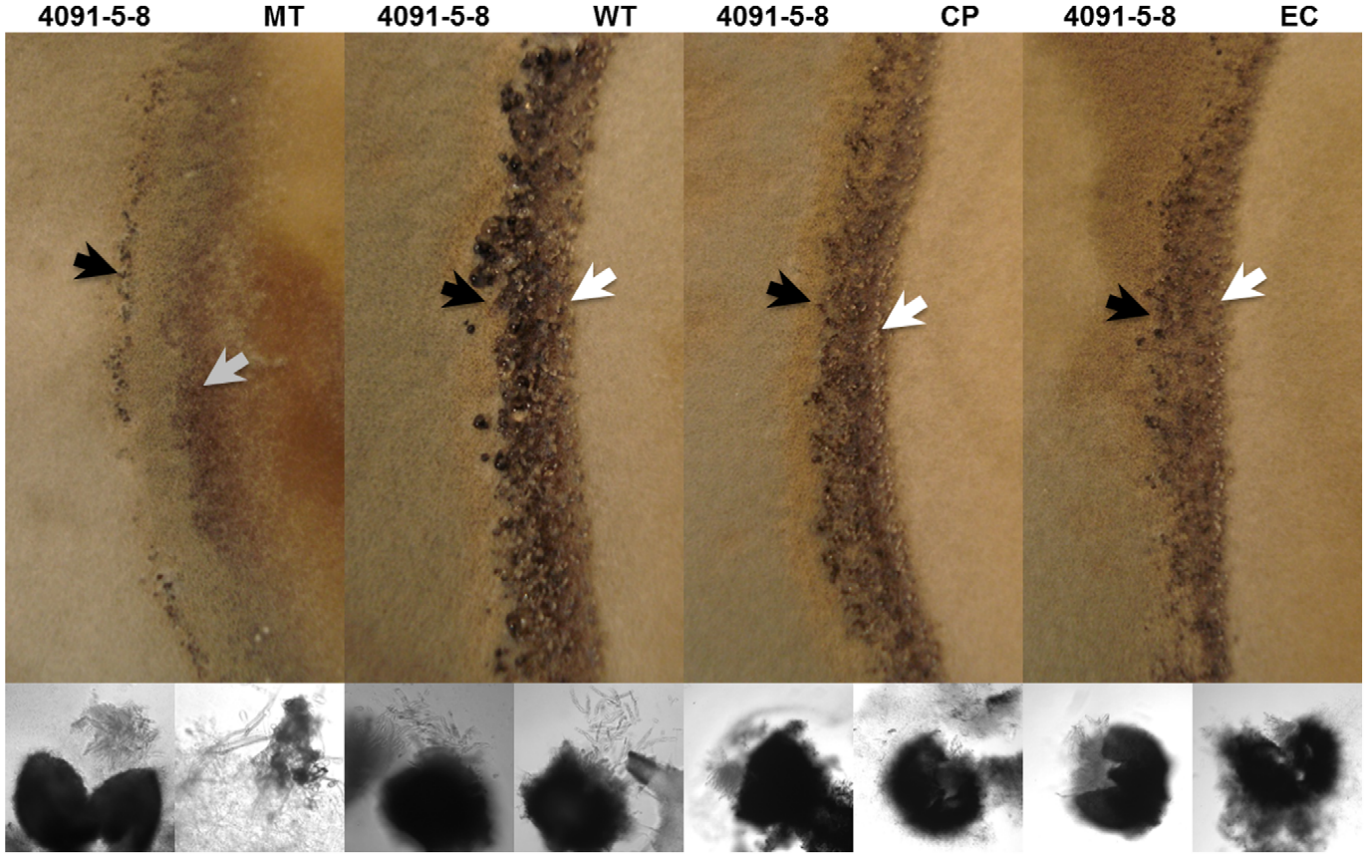
MGG_01282 deletion mutants are female sterile. 70-15 wild type (WT), MGG_01282 deletion mutant (MT), ectopic (EC) and MGG_01282 complemented (CP) strains were crossed with the opposite mating type, 4091-5-8 strain, on oat meal media. Fruiting bodies formed in each test strain and 4091-5-8 strain are marked with white and black arrows respectively. Below are shown corresponding perithecia and erupting ascospores. The melanized structure in MT is indicated by a gray arrow with corresponding enlargement below.

In previous work, we conducted extensive microarray analysis [13], [19]. Further inspection of the data revealed that 161 of the 183 ubiquitin pathway genes were expressed during conidia germination, appressorium formation or nitrogen starvation (Figure 2, Table S2). The most dramatic changes in gene expression occurred during conidia germination, where 87.6% of the genes (141 out of 161) showed at least a 50% increase in transcript levels and 39% were significantly induced (>2 fold up, p<0.05). This is in contrast to the entire transcriptome, where only 21% (2,087 of 10,176) of genes were significantly (>2 fold up, p<0.05) induced. Sixty three percent (5 out of 8) of genes encoding ubiquitin-activating enzyme, E1, 42% (8 out of 19) of ubiquitin-conjugating enzyme, E2, 29% of (23 out of 79) ubiquitin ligase, E3 and 39% (7 out of 18) of de-ubiquitinating enzyme showed increased gene expression. No ubiquitin pathway genes exhibited significant down regulation during conidia germination, except for MGG_01282. Transcripts of MGG_01282, predicted to encode a polyubiquitin protein, were significantly more abundant in intact conidia compared with germinating cells.

Contrary to the conidia germination, transcript levels of the majority of ubiquitin associated proteins did not change significantly during appressorium formation. Only 3.7% (6) and 5.0% (8) of genes were induced during appressorium induction in response to hydrophobic surface signal and cyclic AMP, respectively, and 0.6% (1) and 2.5% (4) genes were down regulated. Transcripts of MGG_01282, a polyubiquitin protein and MGG_07127, an autophagy-related E1 like protein were more abundant during appressorium formation compared to germinating conidia. No ubiquitin-conjugating enzyme, E2, genes were differentially expressed. Among 79 ubiquitin ligases, E3, 4% (3) and 8% (6) genes were induced by physical and chemical signals and only one gene, MGG_10932 was down regulated. Two F-box proteins, MGG_07785 and MGG_08019, were up regulated by both signals. MGG_08638, a ubiquitin C-terminal hydrolase was the sole de-ubiquitination gene that was induced by the hydrophobic physical cue.

Examination of previous data under nitrogen starvation conditions revealed transcriptional induction of about 9% (14) and 11% (18) of ubiquitin pathway genes, and 3% (4) and 2% (3) with reduced expression of 12 hr and 48 hr, respectively. Two F-box containing proteins (MGG_00768 and MGG_04395) and one zinc finger, ring-type E3 ligase (MGG_02837) were induced at both 12 hr and 48 hr. The expression of only 2 genes was up-regulated by appressorium inducing physical and chemical signals as well as by nitrogen starvation. Transcript levels of MGG_01282 were significantly elevated during appressorium maturation (3.7 fold) and cyclic AMP induction (3.0 fold) as well as with nitrogen starvation for 12 hr (8.2 fold) and 48 hr (2.4 fold). Expression of the F-box protein, MGG_07785 was induced during appressorium formation and at 12 hr nitrogen starvation. In sum, transcription levels of a large proportion (39%) of genes associated with the ubiquitination pathway were induced during conidia germination. However, only a small proportion (between 5–11%) was induced during appressorium formation or in response to nitrogen starvation. Very few genes were induced under both conditions. One of the notable exceptions was the gene encoding polyubiquitin, MGG_01282.

### Highly Conserved but Structurally Diverse Ubiquitin Genes in M. oryzae Show Different Expression Patterns during Development and Starvation

In addition to MGG_01282, which encodes a protein with 4 ubiquitin monomers, we identified 11 other proteins in *M. oryzae* that contain single or partial ubiquitin motifs. Some of which contained other functional domains that are related to the ubiquitination pathway (Figure 3A). MGG_09887 encodes a single ubiquitin monomer, whereas other single ubiquitin proteins MGG_06044 and MGG_07928 contain large and small ribosomal subunits at their C terminals, respectively. Among the proteins that contain a partial ubiquitin moiety, MGG_01318 and MGG_01656 possess an ubiquitin associated domain and MGG_02970 has a ubiquitin carboxyl-terminal hydrolase domain at the C terminal region. Two proteins, MGG_05137 and MGG_05737 contain an ubiquitin-like SUMO domain at their C terminal and N terminal regions, respectively.

Except for MGG_01282, expression of all ubiquitin containing genes were induced during conidia germination, however, no significant change was observed during appressorium induction and nitrogen starvation in most cases (Figure 3). The exceptions were MGG_07928 and MGG_06044, which were down-regulated during both conditions. Gene expression of MGG_09887, encoding a single ubiquitin moiety, was induced under nitrogen starvation. Because MGG_01282 showed the most dynamic changes in gene expression, being highly induced during appressorium formation and nitrogen starvation, it was subjected to further examination.

### Nitrogen Starvation Results in a Dramatic Increase in Protein Ubiquitination which is Mediated by the Polyubiquitin Protein, MGG_01282

Protein ubiquitination is known to be directly linked to the cellular nutrient status [25]. To examine this relationship in *M. oryzae*, we investigated the correlation between ubiquitination and one of the major developmental signals, nitrogen starvation. As shown by immunoblots with antibodies recognizing polyubiquitin and ubiquitin in Figure 4A, when the fungus was exposed to the nitrogen limiting conditions for 12 hr, protein ubiquitination dramatically increased compared to the condition without nitrogen stress. To further investigate the function of MGG_01282, we generated deletion mutants using standard protocols [19]. Immunoblot analyses of proteins extracted from growth on minimal medium or under nitrogen starvation showed mutant strains contained strikingly less ubiquitinated proteins compared to wild type and ectopic strains (Figure 4B). In addition, immunoblot analyses using the linkage specific antibody (K63) revealed mgg_01282 deletion mutants contained dramatically reduced K63 linked polyubiquitin targets. K63 ubiquitin targets are known to be involved in cellular development and signal transduction in eukaryotic cells [26]. In summary, protein ubiquitination was significantly elevated under nitrogen starvation and was primarily mediated by polyubiquitin protein MGG_01282.

### The Polyubiquitin Protein MGG_01282 is Essential for Fungal Growth, Development and Pathogenicity

To investigate the biological role of polyubiquitin in *M. oryzae*, we compared phenotypes of the knockout mutant with ectopic and wild type strains as well as with a strain complemented with MGG_01282. Deletion of MGG_01282 resulted in significant changes in fungal growth, morphology and development in *M. oryzae*. Radial growth of mutants on solid nutrient rich complete medium or minimal medium was significantly retarded (about 17%) and aerial hyphae was suppressed compared with the wild type. This growth reduction was more severe under nitrogen limiting conditions (Figure 5A and 5B).

MGG_01282 mutants also showed defects in conidiation. The number of conidia generated by the mutants after 7 day incubation on V8 medium was significantly reduced (more than 90%) compared to the wild type (Figure 6). In addition, while most conidia (≥94%) from wild type consisted of three cells, divided by two septa, about half of mutant conidia contained single (35%) or no septa (13%) (Figure 7A). Mutant conidia were smaller and more rounded than those produced by the wild type strain (Figure 7B). Ectopic and complemented strains were indistinguishable from the wild type for growth, conidiation and conidia morphology (Figures 5, 6, and 7).

Almost all wild type and ectopic conidia germinated on hydrophobic surface and about 90% of them successfully developed appressoria after 24 hr incubation. In mutant strains, 62% of conidia germinated but most failed to develop appressoria (Figure 8A and 8B). In addition, mutant appressoria were less pigmented and unable to cause disease. When sprayed on barley and rice leaves, mutant strains produced no disease symptoms (Figure 8C). To investigate whether MGG_01282 mediated growth in planta, barley leaves were inoculated onto pinprick wounded sites. MGG_01282 deletion mutants showed no blast symptoms on wounded plants (Figure 8D). Taken together, these data indicate that the polyubiquitin gene, MGG_01282 is essential for conidiation, appressorium formation and invasive growth in planta.

*M. oryzae* 70-15 is heterothallic and requires an opposite mating type strain for sexual reproduction. In this study, we crossed the wild type 70-15, MGG_01282 deletion mutant, ectopic and complemented strains with strain 4091-5-8. Four weeks after crossing, wild type, ectopic and complemented strains developed melanized perithecia, which contained ascoconidia within the asci (Figure 9). In these crosses, all strains were female fertile and two rows of perithecia were clearly visible (Figure 9). In crosses with MGG_01282 deletion mutant, the number of perithecia was reduced. Within 4091-5-8 tissue, typical asci were observed. However, within the deletion mutant tissue only highly condensed and melanized fungal structures were detected that failed to further develop into perithecia (Figure 9). These data indicate that MGG_01282 is required for normal sexual development.

### Ubiquitinated Protein Profiling during Fungal Growth Under Nitrogen Stress

We found that protein ubiquitination is highly increased during nitrogen starvation. This suggests that when nitrogen is limiting, *M. oryzae* actively recycles cellular materials and redirects biological pathways to cope with the nitrogen deficiency for survival, at least part of which is mediated by protein ubiquitination. To identify proteins targeted for ubiquitination under nitrogen stress, we incubated wild type *M. oryzae* in minimal media without a nitrogen source for 18 hr and enriched polyubiquitinated proteins using agarose TUBE2. TUBE2 contains a protein with very high affinity for polyubiquitinated proteins [27]. As a negative control, we used agarose lacking the affinity protein. We found the enrichment to be effective as evidenced by protein staining and immunoblot analysis of the enriched protein fraction (Figure S2). Mass spectral analysis revealed 63 proteins to be unique or significantly enriched in the affinity purified sample compared to negative control (Table S3). As expected, ubiquitin was found to be the most abundant protein based on spectral counts. Based on gene ontology using BLAST2GO [28], compared to the entire proteome, proteins involved in translation (30.2%), metabolic process (17.4%), transport (14.3%), and protein metabolic process (9.5%) including ubiquitin proteins and components of the proteasome were over-represented (Table S3). Several proteins associated with cytoskeleton and stress responses including actin, tubulins and heat shock proteins were also identified (Table S3). We further identified proteins predicted to be involved repression of carbon catabolism such as homologs of a hulA E3 ligase (MGG_07255) and as arrestin containing protein (MGG_01045) as well as proteins involved in cell signaling including MGG_01588 and MGG_13806, both 14-3-3 proteins.

## Discussion

The availability of the entire genome sequence and global gene expression profiles for *M. oryzae* has enabled new insight into infection related development and pathogenicity [19], [29]. Our previous studies revealed a hitherto unknown link between protein degradation and infection structure development in *M. oryzae* [19]. Genes required for the non-selective protein degradation process referred to as autophagy have been characterized and shown to be important for fungal pathogenicity in *M. oryzae* [30], [31]. On the other hand, prior to this study, little direct evidence was available linking the highly selective protein degradation process, mediated by ubiquitin, to fungal pathogenicity.

To establish a link between protein turnover through the ubiquitin-proteasome complex and fungal pathogenicity, we first demonstrated that treatment with the 26S proteasome inhibitor, Bortezomib, resulted in a significant delay in germination and appressorium formation. Moreover, addition of Bortezomib to inoculum blocked symptom development, even though melanized appressoria were formed after 48 hr. A recent report, which was published during the course of this research, also showed the proteasome inhibitors MG-132, proteasome inhibitor I and proteome inhibitor II delayed conidia germination and appressorium formation in *M. oryzae* as well as suppressed infection of rice leaves [32].

In order to elucidate the underlying mechanisms, we then explored the machinery associated with ubiquitin mediated protein turnover. Similar to other eukaryotic organisms [20], many of the components are highly conserved in *M. oryzae*. Examination of whole genome microarray gene expression data further revealed that most of the components for protein ubiquitination were induced upon conidia germination but did not dramatically change expression during appressorium formation. The elevated expression of genes associated with ubiquitination during germination in the absence of external nutrients likely reflects the cells preparation for recycling proteins and other storage components. A few genes were observed to be induced during appressorium induction and nitrogen starvation but none of them had been previously characterized. Interestingly, we discovered that a polyubiquitin encoding gene MGG_01282 was most highly expressed in intact conidia rather than in germinating conidia and was significantly induced during appressorium formation and nitrogen starvation.

In fungi, polyubiquitin does not appear to be an essential gene, although it likely provides the main supply of cellular ubiquitin protein in response to developmental and environmental stimuli. Here, we showed by Western blot analysis that protein ubiquitination is highly induced under nitrogen starvation and through examination of the knock-out mutant that protein ubiquitination is mainly mediated by MGG_01282 gene products. The gene deletion mutant in *M. oryzae*, although viable, exhibited numerous phenotypic detects, including defects in mycelia growth, conidia morphology, sexual reproduction, infection structure development and pathogenicity. Similar types of defects have been observed in *Saccharomyces cerevisiae* and *C. albicans*. Loss of *UBI4* in *S. cerevisiae* resulted in increased sensitivity to starvation and to amino acid analogs as well as reduced growth at high temperatures [33]–[35]. *UBI4* deletion mutant of *C. albicans* grew relatively normally on rich media, but displayed morphological and cell cycle defects when exposed to a number of stresses including temperature, peroxide and several anti-fungal drugs that interfere with cell wall biosynthesis [9]. In *M. oryzae,* the polyubiquitin deletion mutant although growing relatively poorly on all media, was most affected under nitrogen starvation. These data are consistent with ubiquitination playing a major role in protein turnover required for normal growth and development under a variety of stress conditions.

Examination of the 63 proteins unique or significantly enriched following affinity purification for polyubiquitination revealed that the most abundant group of proteins was components of the ribosome. Other studies have also shown that proteins associated with ribosome are ubiquitinated [9], [36]. As may be expected under nitrogen starvation, translation is curtailed and the machinery is recycled via ubiquitination and proteasome mediated degradation. We also identified other proteins including heat shock proteins and a succinate dehydrogenase, which in *C. albicans* showed increased ubiquitination in response to heat and oxidative stress [9]. In a global analysis of ubiquitination in the human cell line HEK293, 236 proteins were identified to be ubiquitinated [37]. Among these, we found 20 that matched *M. oryzae* proteins in our ubiquitinated protein data set and these included heat shock proteins, actin and tubulins and ribosomal proteins. These data suggest a certain level of conservation of proteins targeted for ubiquitination across kingdoms.

It was noteworthy that two of the proteins targeted by ubiquitin identified in our data set were a HECT domain containing E3 ligase, MGG_07255 and an arrestin domain containing protein MGG_01045, orthologs of Rsp5 and Rod1 in *S. cerevisiae* respectively. Rsp5 is known to be involved in a variety of cellular process including endocytosis, multivesicular body sorting and RNA stability [38]–[40]. The arrestin-like adaptor, Rod1 binds to Rsp5 and mediates ubiquitination and endocytic internalization of membrane transporters, which are degraded in the vacuole. In *A nidulans,* CreD, an ortholog of Rod1, has been shown to control carbon catabolite repression with possible interaction with HECT ligase, HulA [41]. Self-ubiquitination of other HECT ligase ortholog NEDD4-1, NEDD4-2 is regulated through intramolecular interaction between the WW domains and PY motifs in HECT domains [42], [43]. This suggests that ubiquitination and following degradation of MGG_07255 is tightly regulated depending upon environmental conditions. During nitrogen starvation, ubiquitination of MGG_07255/MGG_01045 could be important for reprogramming fungal cells to cope with nutrient limiting conditions.

Over the past twenty years, the role of protein ubiquitination in eukaryotic cells has been emerging, however, relatively little is known related to fungal development, pathogenicity and disease control. Here, we showed through a combination of pharmacological, molecular and proteomic analysis that ubiquitin mediated posttranslational modification is a central regulator in fungal nutrition, development and pathogenicity. Future study will be focused on specific components of the ubiquitination processes including identification of specific ubiquitination target proteins, which may offer up novel strategies for plant disease control.

## Supporting Information

Figure S1 **Targeted knock out of the polyubiquitin gene (MGG_01282) in** ***M. oryzae*** **.** A. A gene replacement cassette in which the hygromycin resistance gene (HPH) was flanked with about 1 kb each upstream and downstream of the MGG_01282 gene was constructed and transformed into wild type cells. Through homologous recombination, the MGG_01282 gene was replaced with HPH generating MGG_01282 deletion mutants. B. Deletion mutants were confirmed by Southern blot analysis using right flanking sequences (*) as a probe. Absence of Xho I restriction enzyme recognition site (X) in HPH resulted in a larger labeled fragment in the mutant which was distinct from that of wild type.
(TIF)

Figure S2 **Enrichment of ubiquitinated proteins using TUBE2 (Tandem Ubiquitin Binding Entity 2).** 85 ug of *M. oryzae* total proteins were extracted from liquid minimal media without nitrogen sources and was subjected to pulldown with Agarose-TUBE2. Input (I), unbound(U), eluted (E) and wash (W) samples were load to 4–20% gradient gel (Invitrogen) along with protein marker(M). Following electrophoresis, gel was stained with coomassie blue. After blotting, membrane was stained with ponceau and was probed with anti-ubiquitin antibody. Compared to the amount of total proteins, ubiquitinated proteins were found to be highly enriched in the eluted sample.
(TIF)

Table S1 **Primer sequences for gene specific replacement cassette construction.**
(XLS)

Table S2 **List of ubiquitin pathway associated proteins in** ***M.oryzae*** **and gene expression profiling during conidia germination, appressorium formation and under nitrogen starvation.**
(XLSX)

Table S3 **List of putative ubiquitinated proteins during nitrogen starvation in** ***M. oryzae*** **.**
(XLSX)
