# Supplementary figures and images for: Polyubiquitin Is Required for Growth, Development and Pathogenicity in the Rice Blast Fungus Magnaporthe oryzae
Source: PLoS One. 2012 Aug 10;7(8):e42868. doi: 10.1371/journal.pone.0042868 (PMC3416782; doi:10.1371/journal.pone.0042868)

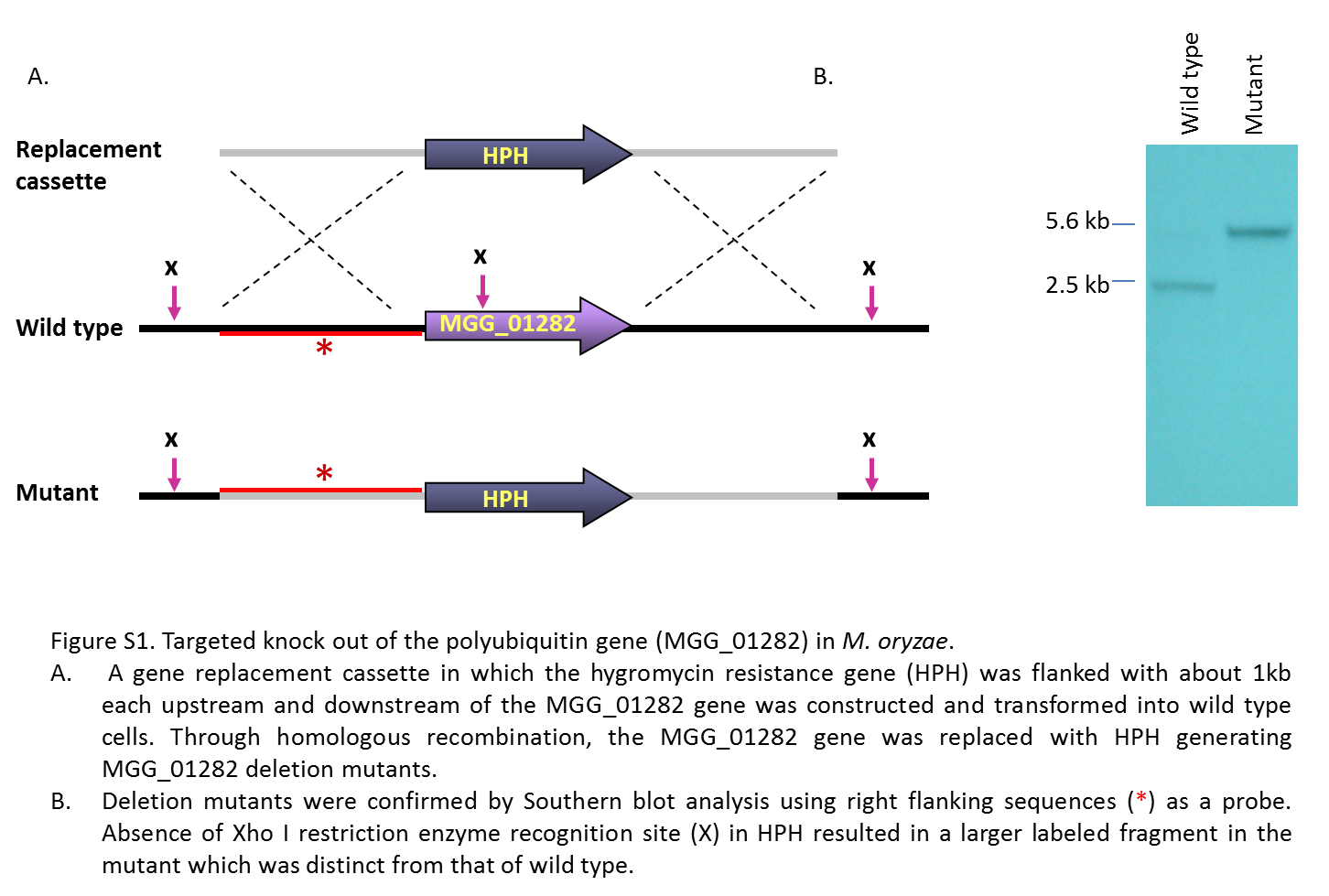

Supplement: Figure S1 — Targeted knock out of the polyubiquitin gene (MGG_01282) in M. oryzae . A. A gene replacement cassette in which the hygromycin resistance gene (HPH) was flanked with about 1 kb each upstream and downstream of the MGG_01282 gene was constructed and transformed into wild type cells. Through homologous recombination, the MGG_01282 gene was replaced with HPH generating MGG_01282 deletion mutants. B. Deletion mutants were confirmed by Southern blot analysis using right flanking sequences (*) as a probe. Absence of Xho I restriction enzyme recognition site (X) in HPH resulted in a larger labeled fragment in the mutant which was distinct from that of wild type. (TIF) [file pone.0042868.s001.tif]

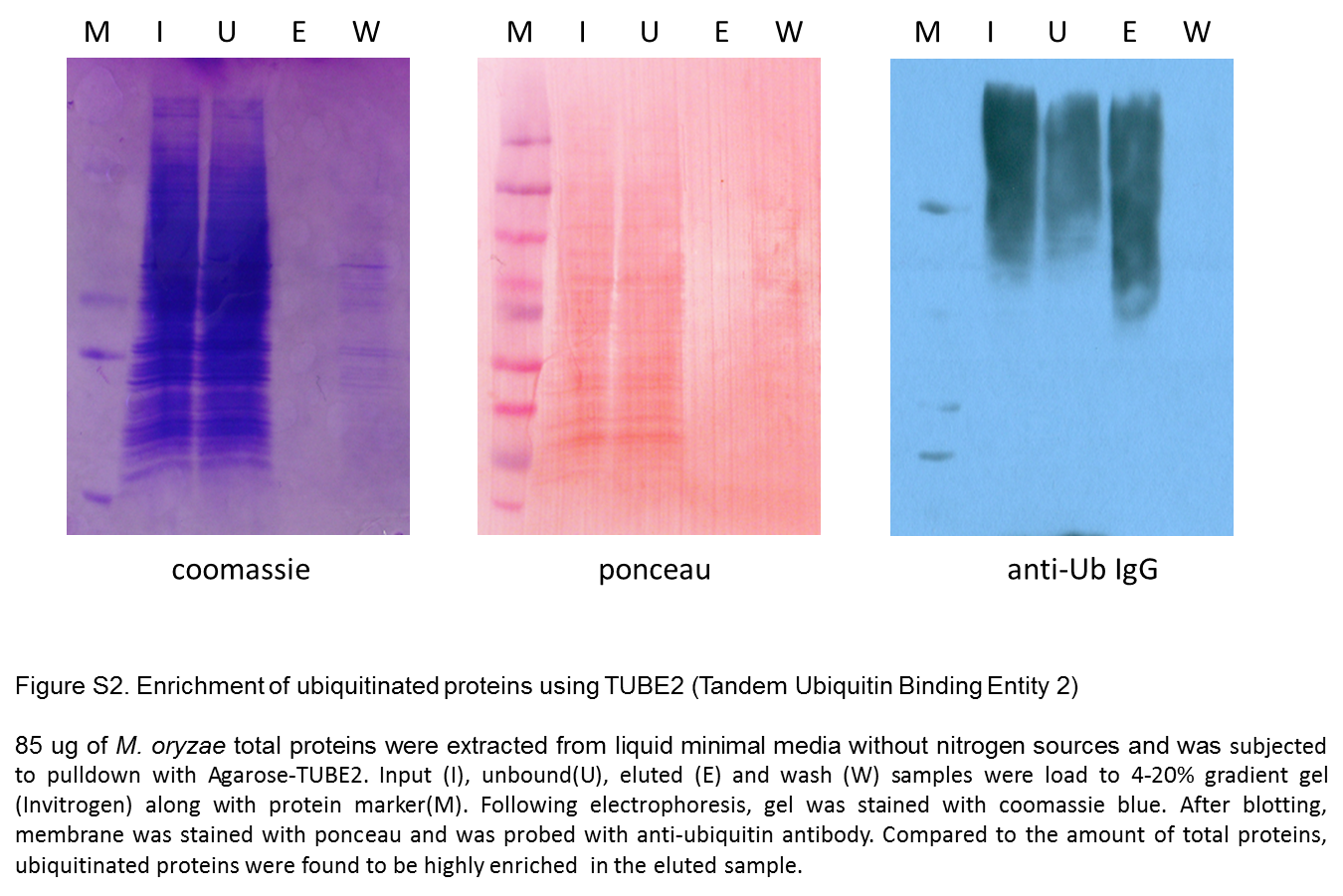

Supplement: Figure S2 — Enrichment of ubiquitinated proteins using TUBE2 (Tandem Ubiquitin Binding Entity 2). 85 ug of M. oryzae total proteins were extracted from liquid minimal media without nitrogen sources and was subjected to pulldown with Agarose-TUBE2. Input (I), unbound(U), eluted (E) and wash (W) samples were load to 4–20% gradient gel (Invitrogen) along with protein marker(M). Following electrophoresis, gel was stained with coomassie blue. After blotting, membrane was stained with ponceau and was probed with anti-ubiquitin antibody. Compared to the amount of total proteins, ubiquitinated proteins were found to be highly enriched in the eluted sample. (TIF) [file pone.0042868.s002.tif]
